# Association between pain sensitivity and gray matter properties in the sensorimotor network in women with irritable bowel syndrome

**DOI:** 10.1111/nmo.14027

**Published:** 2020-11-10

**Authors:** Cecilia Grinsvall, Hyo Jin Ryu, Lukas Van Oudenhove, Jennifer S. Labus, Arpana Gupta, Maria Ljungberg, Hans Törnblom, Emeran A. Mayer, Magnus Simrén

**Affiliations:** ^1^ Department of Internal Medicine & Clinical Nutrition Institute of Medicine Sahlgrenska Academy University of Gothenburg Gothenburg Sweden; ^2^ Vatche and Tamar Manoukian Division of Digestive Diseases David Geffen School at UCLA Los Angeles CA USA; ^3^ Translational Research Center for Gastrointestinal Disorders (TARGID) KU Leuven Leuven Belgium; ^4^ Department of Radiation Physics Institute of Clinical Sciences Sahlgrenska Academy University of Gothenburg Gothenburg Sweden; ^5^ Department of Medical Physics and Biomedical Engineering Diagnostic Imaging Sahlgrenska University Hospital MR Centre Gothenburg Sweden; ^6^ Center for Functional Gastrointestinal and Motility Disorders University of North Carolina at Chapel Hill Chapel Hill NC USA

**Keywords:** gray matter morphometry, irritable bowel syndrome, structural brain imaging, visceral sensitivity

## Abstract

**Background:**

Enhanced perception of visceral stimuli is an important feature of Irritable Bowel Syndrome (IBS), but it is not known whether visceral sensitivity is associated with regional structural brain properties in IBS.

**Methods:**

Structural brain magnetic resonance imaging data from 216 women with IBS and 138 healthy women were parcellated with FreeSurfer to define regional gray matter morphometry (volume, cortical thickness, surface area and mean curvature) in the sensorimotor network. General linear models were used to detect group differences between IBS and health. In a second set of 48 female IBS patients, pain threshold, pain intensity ratings during rectal balloon distension, and reported levels of abdominal pain and bloating were correlated with brain regions that showed differences between IBS and health in the first data set.

**Key Results:**

Several statistically significant differences between IBS patients and healthy controls were found, mainly higher gray matter volume and cortical thickness in primary somatosensory cortex, secondary somatosensory cortex, and subcortical regions, and lesser gray matter volume, surface area and cortical thickness in posterior insula and superior frontal gyrus. Pain intensity ratings during rectal distension were associated with left primary somatosensory cortical thickness, and pain threshold was associated with right nucleus accumbens volume.

**Conclusions and Inferences:**

Regional gray matter differences in sensorimotor network are associated with visceral sensitivity and may represent neuroplastic changes in female IBS patients.

## Graphical abstract

Regional gray matter differences in sensorimotor network were identified between female IBS patients and female healthy controls. Some regions were associated with visceral sensitivity in IBS and may represent neuroplastic changes in female IBS patients.

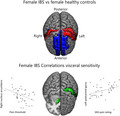

**Key points**

- Visceral sensitivity is of importance for IBS, and regional gray matter differences between IBS and health have been shown. There is no previous study investigating possible regional gray matter morphometry associations with visceral sensitivity in IBS.
- Right nucleus accumbens volume correlated with pain threshold, and left primary somatosensory cortical thickness correlated with pain intensity ratings during rectal distension.
- Regional gray matter differences in sensorimotor network were partly associated with visceral sensitivity and may represent neuroplastic changes in female IBS patients.

## 1 INTRODUCTION

Irritable bowel syndrome (IBS) is one of the most common disorders of gut‐brain interaction.^1^ It is characterized by chronic, recurrent abdominal pain, and altered bowel habits^2^ and is often accompanied by comorbid psychiatric and other chronic pain disorders.^3^ Visceral hypersensitivity has consistently been identified as an important feature of IBS.^4^ Dysregulation of the brain‐gut axis^5^ and in particular aberrant central sensory processing^6^, ^7^, ^8^ have been found to be contributing mechanisms in IBS, although they remain incompletely understood. Among the proposed central networks relevant to IBS pathophysiology, the sensorimotor network has shown structural and functional differences between healthy controls (HCs) and IBS patients across several studies.^9^, ^10^, ^11^ The sensorimotor network includes primary and secondary sensorimotor cortex, primary and supplementary motor cortex, posterior insula, basal ganglia, and thalamus.^9^ Higher gray matter volume of the primary somatosensory cortex has been reported in IBS,^12^ as well as in conditions such as interstitial cystitis and painful bladder syndrome.^13^ Furthermore, the literature supports positive associations of cortical thickness in the primary sensorimotor regions with gastrointestinal symptom severity, whereas right anterior and middle insular cortex has shown negative associations with anxiety in women with IBS.^14^ While evoked visceral pain has shown associations with regional gray matter properties in HCs^15^ and resting state functional connectivity in IBS,^11^ correlations of evoked visceral pain using rectal balloon distension and regional gray matter morphometric properties in IBS have not been reported previously.

In the current study, we aimed to identify the relationship between sensorimotor network gray matter morphometry and evoked and spontaneous pain measures in women with IBS. First, we assessed differences in the gray matter properties of sensorimotor network between IBS and HCs in a large collection of subjects recruited from different studies. Thereafter, we examined correlations of these regional brain measurements in a second set of women with IBS with two types of pain assessments: experimentally evoked rectal pain and reported symptom severity.

Our hypothesis was that evoked pain, spontaneous symptoms of sensory origin, and psychological distress would show different associations with gray matter morphometry in regions of the sensorimotor network in IBS. More specifically, we hypothesized that symptoms of abdominal pain and bloating would show correlations with primary motor cortex and primary somatosensory cortex^14^; visceral sensitivity measures during rectal barostat would show correlations with posterior insula^11^; psychological distress would show no associations as it was previously shown to be more relevant to emotional‐arousal, salience, and central autonomic networks than the sensorimotor network.^8^

## 2 METHODS AND MATERIALS

### 2.1 Dataset 1: Analysis of a large archived brain imaging dataset

#### 2.1.1 Subjects and brain imaging

Structural brain images from IBS and HCs were obtained from the NIH‐funded Pain and Interoception Imaging Network (PAIN).^16^ Parts of the dataset have been studied and published previously, references^10^, ^12^, ^14^ are of relevance to the present study. The brain images were acquired using a 3.0 T Siemens Trio MRI (Siemens Healthineers AG, Erlangen, Germany) at the G. Oppenheimer Center for Neurobiology of Stress and Resilience at the University of California, Los Angeles between 2006 and 2017. IBS diagnoses were based on Rome III or Rome IV criteria depending on time of inclusion. Structural scans were obtained from six different acquisition sequences using a high‐resolution 3‐dimensional T1‐weighted, sagittal magnetization‐prepared rapid gradient echo protocol as described in Table 1. Acquisition protocols were only included if they were used with both IBS and HCs. A general linear model controlling for age indicated that protocol 1 and 2 were similar to each other but had lower total gray matter volumes than the remaining protocols. Fisher's two‐sided exact test indicated that the distribution of groups across the protocols was significantly different, *p* = 0.05 (Protocol 1: 33 HCs, 33 IBS; Protocol 2: 105 HCs, 183 IBS). As such, we controlled for protocol in the subsequent statistical analyses.

**TABLE 1:** Structural acquisition protocols

Protocol	TR	TE	FA	VOX
P001	2200	3.26	9	1 × 1 × 1
P002	2200	3.27	9	.9 × .9 × 1
P003	20	3	25	1 × 1 × 1
P004	2200	3.26	20	1 × 1 × 1
P005	2300	2.98	9	1 × 1 × 1
P006	2300	2.85	9	1 × 1 × 1

Abbreviations: FA, flip angle; TE, echo time (ms); TR, repetition time (ms); VOX, voxel size (mm).

#### 2.1.2 Structural MRI analysis

**FIGURE 1:**
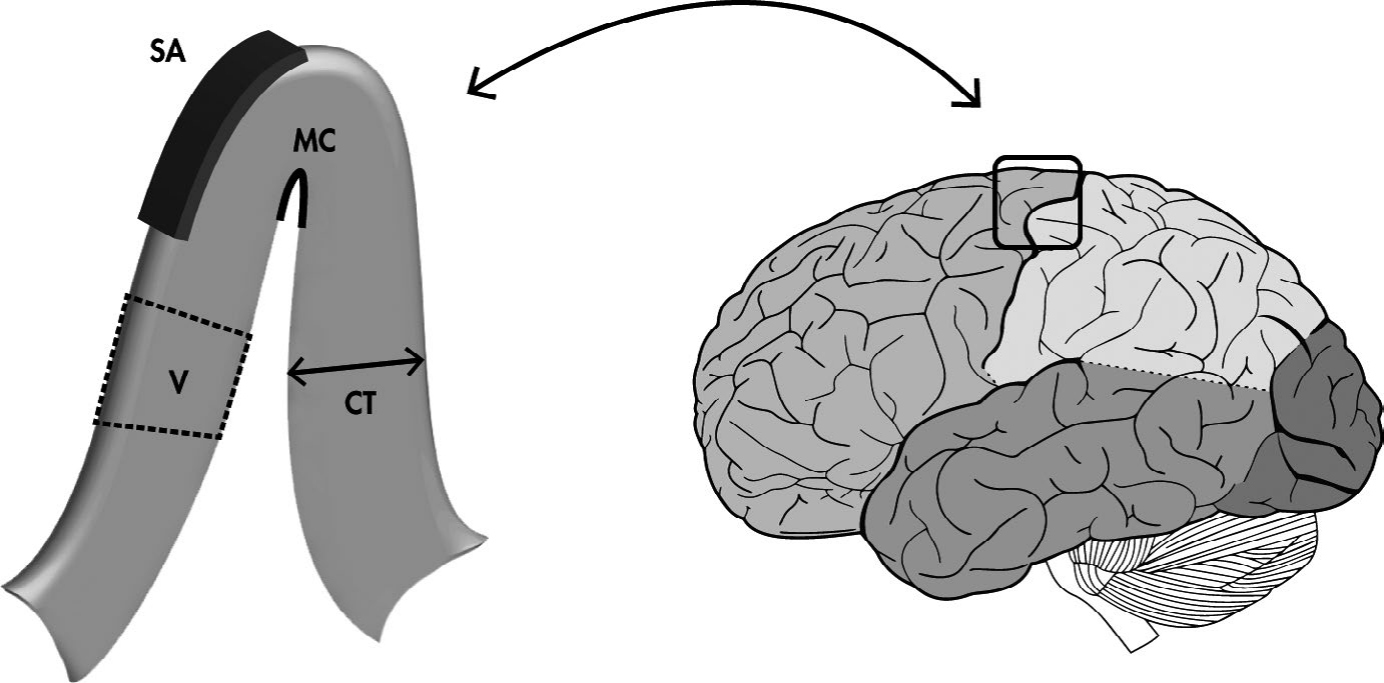
A graphical representation of the gray matter morphometric measurements. CT, cortical thickness; MC, mean curvature; SA, surface area; V, volume

After quality control, FreeSurfer^17^, ^18^ was used for brain segmentation and parcellation of regions of interest based on the Destrieux atlas and Harvard‐Oxford subcortical atlas^19^, ^20^, ^21^ using the recon‐all command. Quality control was based on various indicators of scan quality, including the absence of severe noise and artifacts and correct segmentation of gray and white matter. Any scans with major pathologies or errors that were deemed sufficiently harmful to structural measures were omitted from further analyses. Four morphological measures were computed for each cortical parcellation representing distinct features of the cortex: volume (V), surface area (SA), cortical thickness (CT), and mean curvature (MC) (Figure 1). For subcortical structures, only volume was computed.

#### 2.1.3 Regions of interest (ROIs)

Regions comprising the sensorimotor network includes thalamus, basal ganglia the posterior insula, and the primary and secondary sensorimotor cortices.^9^ In total, 34 regions of interest (17 ROIs, 2 hemispheres) were examined (see Table 2).

**TABLE 2:** ROIs in the sensorimotor network

Hemisphere	Short name	Description	Subregion
Left and Right	Tha	Thalamus	Thalamus
Left and right	CaN	Caudate nucleus	Basal Ganglia
Left and right	Nacc	Nucleus accumbens	Basal Ganglia
Left and right	Pal	Pallidum	Basal Ganglia
Left and right	Pu	Putamen	Basal Ganglia
Left and right	PosCS	Postcentral sulcus	S1
Left and right	CS	Central sulcus (Rolando's fissure)	S1
Left and right	PosCG	Postcentral gyrus	S1
Left and right	SbCG_S	Subcentral gyrus (central operculum) and sulci	S2
Left and right	InfCirIns	Inferior segment of the circular sulcus of the insula	pINS
Left and right	LoInG_CInS	Long insular gyrus and central sulcus of the insula	pINS
Left and right	PosLS	Posterior ramus of the lateral sulcus	pINS
Left and right	PRCG	Precentral gyrus	M1
Left and right	InfPrCS	Inferior part of the precentral sulcus	M1
Left and right	SupPrCs	Superior part of the precentral sulcus	M1
Left and right	SupFG	Superior frontal gyrus	M2/SMA
Left and right	SupFS	Superior frontal sulcus	M2/SMA

Abbreviations: M1, primary motor cortex; M2/SMA, supplementary motor area; pINS, posterior insula; S1, primary somatosensory cortex; S2, secondary somatosensory cortex.

#### 2.1.4 Statistical analysis of Dataset 1

Using Matlab, R2017b, contrast analysis within the framework of the general linear model (GLM) was used to detect group differences in gray matter morphometry between IBS and HCs, controlling for age, total intracranial volume, and acquisition protocol. ROIs demonstrating significant group differences (*p* < 0.05, Bonferroni corrected for the number of ROIs tested) were used as ROIs for correlational analysis in a second independent data, Dataset 2.

### 2.2 Dataset 2: Analysis of a well‐characterized IBS cohort

#### 2.2.1 Subjects

Female IBS patients were recruited at the gastroenterology outpatient clinic specializing in functional GI disorders at Sahlgrenska University Hospital in Gothenburg, Sweden, between 2011 and 2014. The IBS patients (18–65 years) were enrolled through self‐referral or referral by other physicians, mostly primary care doctors. The diagnosis was based on clinical presentation, fulfillment of the Rome III criteria for IBS,^22^ and additional investigations if considered necessary by the gastroenterologist (HT or MS). Exclusion criteria included abnormal results on standard screening laboratory tests, severe psychiatric, systemic, or other GI diseases, history of drug or alcohol abuse, and the inability to reliably respond to questionnaires in Swedish. The use of drugs affecting GI function, anti‐ or probiotics were not allowed during the study period, and contraindications for MRI scanning were screened for. All patients gave their written consent to participate after verbal and written information. The study protocol was approved by the Regional Ethical Review Board in Gothenburg prior to the start of patient inclusion. The work in this article has been carried out in accordance with The Code of Ethics of the World Medical Association (Declaration of Helsinki).

#### 2.2.2 Rectal barostat procedure

The rectal barostat protocol described by Cremonini et al^23^ was used. Briefly, the protocol used a computer‐driven electronic barostat (Dual Drive Barostat, Distender Series II; G&J Electronics Inc, Toronto, ON, Canada), with the baseline operating pressure (BOP) defined as 2 mm Hg above the minimal distension pressure at which respiratory variations were clearly recorded. An ascending method of limits (AML) rectal distension protocol was used, with ramp inflation starting at 0 mm Hg, increasing by steps of 4 mm Hg with 1 min per step to identify sensory thresholds. Only the pain threshold was used in the analyses for this study. After the AML, subjects randomly received fixed phasic distensions at 12, 24, 36, and 48 mm Hg above BOP and were asked to complete visual analogue scale (VAS) ratings for rectal sensations after 30 s of distension. During the phasic distensions, only one distension level above the pain threshold was delivered for ethical reasons. Most participants completed the 12 and 24 mm Hg distension levels, but the 36 and 48 mm Hg distensions were performed in fewer subjects. For this reason, we included VAS ratings for the 24 mm Hg phasic distension, and only the pain intensity rating was used in this study.

#### 2.2.3 Symptom questionnaires

The clinical symptoms of IBS were measured with the IBS severity scoring system (IBS‐SSS)^24^ and the Gastrointestinal Symptom Rating Scale for IBS (GSRS‐IBS).^25^ In this study, we used IBS‐SSS only descriptively, that is to characterize the overall severity in the IBS population. The GSRS‐IBS is a validated questionnaire of gastrointestinal symptom severity in IBS consisting of 13 questions, divided into 5 domains: abdominal pain, bloating, diarrhea, constipation, and satiety. For the present study, we focus specifically on abdominal pain and bloating, since these presumably are lower GI symptoms of sensory origin.

The Hospital Anxiety and Depression scale (HADS) is a questionnaire used to screen for symptoms of depression and anxiety, and assess their severity. The score is usually calculated separately for anxiety and depression, and higher scores reflect more severe symptoms.^26^ The separation of the subscales has been questioned,^27^ and in this study, we used total HADS score as a measurement of overall psychological distress.

#### 2.2.4 Brain imaging acquisition

The brain images were acquired on a 3 Tesla Philips Achieva (Philips Healthcare, Best, the Netherlands) using the standard 8 channel head coil. For the high‐resolution scan, a T1‐weighted 3D TFE gradient echo scan, that is, a magnetization‐prepared rapid acquisition gradient echo (MP‐RAGE) sequence was used, with TR = 7.0 ms, TE = 3.2 ms, flip angle = 9 degrees, and a voxel size of 1 × 1 × 1 mm^3^. The same quality control and structural MRI analysis as described for Dataset 1 was used also in Dataset 2 (see sections 2.1.2 for details).

### 2.3 Statistical analysis

IBM SPSS statistics version 24 was used for all analyses of Dataset 2. Correlations between regional brain measurements and clinical parameters (GSRS‐IBS: abdominal pain and bloating; rectal barostat: pain threshold and pain intensity ratings; HADS total score) were examined. Partial correlations were run, controlling for age and total gray matter volume (TGMV). Symptoms of pain, bloating, and HADS score were normally distributed, and parametric partial correlations were used. Rectal pain threshold was positively skewed, but the distribution could be normalized using a natural logarithmic transformation, the transformed values were used for the partial correlations. The pain ratings during 24 mm Hg rectal distension were non‐normally distributed and could not be normalized after transformation; hence, non‐parametric partial correlations were used for the correlational analyses for this measurement. The significance level for these analyses was set to *p* < 0.05 Bonferroni corrected for the five sets of analyses performed.

## 3 RESULTS

### 3.1 Dataset 1: Subjects

The analyzed population consisted of 138 healthy women and 216 women with IBS. There was no significant difference in age between the two groups (28.8 ± 9.8 vs 30.6 ± 10.0 years; *p* = 0.1). According to the Rome criteria, 83 (38%) had IBS with constipation (IBS‐C), 56 (26%) IBS with diarrhea (IBS‐D), 60 (28%) IBS with mixed bowel habits (IBS‐M), and 7 (8%) IBS unspecified (IBS‐U). The subgrouping was made according to Rome III or Rome IV criteria based on the time of inclusion. No subject was excluded due to poor image quality.

### 3.2 Dataset 2: Subjects

We included 55 female IBS patients. Of these, 7 were excluded because of pathologies found on MRI scan (*n* = 1), another GI disease discovered during the study (*n* = 2), drop‐out (*n* = 3; completed the brain imaging part but not the rectal barostat testing), and use of probiotics (*n* = 1). No subject was excluded due to poor image quality.

The analyzed population hence consisted of 48 female IBS patients, see Table 3 for descriptive statistics. Based on the IBS‐SSS, the majority of the IBS subjects reported severe IBS symptoms; 6 (13%) had mild IBS (IBS‐SSS < 175), 16 (33%) moderate IBS (IBS‐SSS 175–299), and 26 (54%) severe IBS (IBS‐SSS 300–500). Ten of the patients had IBS‐C (21%), 23 had IBS‐D (48%), 6 had IBS‐M (13%), and 9 had IBS‐U (19%). Of the 48 IBS patients, 16 (33.3%) had HAD anxiety score ≥11 and 32 (66.7%) had HAD anxiety score ≥8. Six (12.5%) IBS patients had HAD depression score ≥11 and 13 (27.1%) had HAD depression score ≥8.

**TABLE 3:** Descriptive statistics of Dataset 2, *n* = 48 female IBS patients

Variable	Mean	Standard deviation	Range
Age (years)	33.0	10.3	20–59
Total HADS score	15.4	7.6	4–35
Pain threshold during rectal barostat	25.1	6.9	12–44
VAS pain ratings during rectal barostat 24 mm Hg	43.9	31.7	0–94

### 3.3 Dataset 1: Group differences between IBS and HCs

**FIGURE 2:**
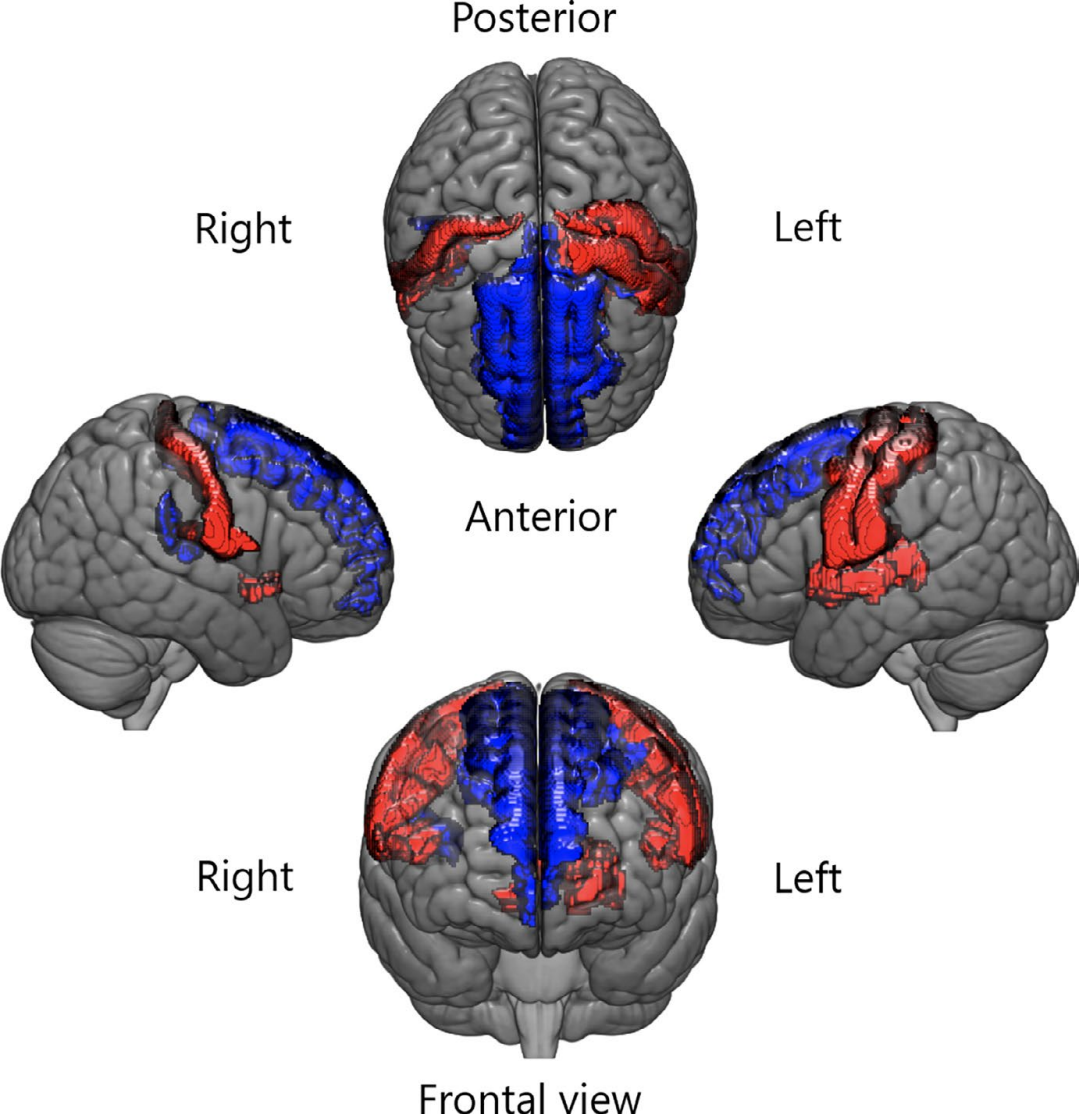
Group differences between IBS patients and HCs in dataset 1. Regions larger in IBS than HCs are shown in red, and regions smaller in IBS than HCs are shown in blue

As shown in Table 4 and Figure 2, differences between IBS and HCs were found in several regions of the sensorimotor network. Specifically, IBS patients had greater cortical thickness in bilateral postcentral gyrus and central sulcus (S1); greater cortical thickness in bilateral, and greater volume in right, subcentral gyrus, and sulcus (S2); and greater cortical thickness and volume of left precentral gyrus (M1). IBS patients also had larger right nucleus accumbens, left putamen, and left thalamus volumes. IBS patients had thinner cortices of bilateral superior frontal gyrus and sulcus (M2/SMA) and smaller volume and surface area of the posterior part of the lateral fissure (pINS).

**TABLE 4:** Group differences between IBS and HCs significant after correcting for multiple comparisons in Dataset 1

Hemisphere	Subregion	Measurement	Region	Result direction	*t*‐value	*p*‐value Bonferroni corrected
Left	Thalamus	Vol	Thalamus	IBS > HCs	−4.02	2.25 × 10^−3^
Left	Putamen	Vol	BG	IBS > HCs	−3.40	2.45 × 10^−2^
Right	Nucleus accumbens	Vol	BG	IBS > HCs	−3.48	1.87 × 10^−2^
Left	Central sulcus	CT	S1	IBS > HCs	−5.37	4.15 × 10^−6^
Right	Central sulcus	CT	S1	IBS > HCs	−3.30	3.54 × 10^−2^
Left	Postcentral gyrus	CT	S1	IBS > HCs	−4.59	1.88 × 10^−4^
Right	Postcentral gyrus	CT	S1	IBS > HCs	−3.70	8.08 × 10^−3^
Left	Subcentral gyrus (central operculum) and sulci	CT	S2	IBS > HCs	−6.08	8.07 × 10^−8^
Left	Subcentral gyrus (central operculum) and sulci	Vol	S2	IBS > HCs	−3.51	1.64 × 10^−2^
Right	Subcentral gyrus (central operculum) and sulci	CT	S2	IBS > HCs	−3.91	3.56 × 10^−3^
Left	Precentral gyrus	CT	M1	IBS > HCs	−4.38	4.85 × 10^−4^
Left	Precentral gyrus	Vol	M1	IBS > HCs	−3.70	8.23 × 10^−3^
Left	Superior part of the precentral sulcus	CT	M1	HCs > IBS	4.19	1.12 × 10^−3^
Right	Posterior part of lateral fissure	SA	pINS	HCs > IBS	3.52	1.61 × 10^−2^
Right	Posterior part of lateral fissure	Vol	pINS	HCs > IBS	3.20	4.97 × 10^−2^
Left	Superior frontal gyrus	CT	M2/SMA	HCs > IBS	4.05	2.01 × 10^−3^
Right	Superior frontal gyrus	CT	M2/SMA	HCs > IBS	5.00	2.74 × 10^−5^
Left	Superior frontal sulcus	CT	M2/SMA	HCs > IBS	3.21	4.78 × 10^−2^
Right	Superior frontal sulcus	CT	M2/SMA	HCs > IBS	4.93	3.88 × 10^−5^

Abbreviations: BG, basal ganglia; CT, cortical thickness; HCs, healthy controls; IBS, irritable bowel syndrome; M1, primary motor cortex; M2/SMA, supplementary motor area; pINS, posterior insula; S1, primary somatosensory cortex; S2, secondary somatosensory cortex; SA, surface area; Vol, Volume.

### 3.4 Dataset 2: Correlations between sensorimotor regions and rectal barostat measurements

**FIGURE 3:**
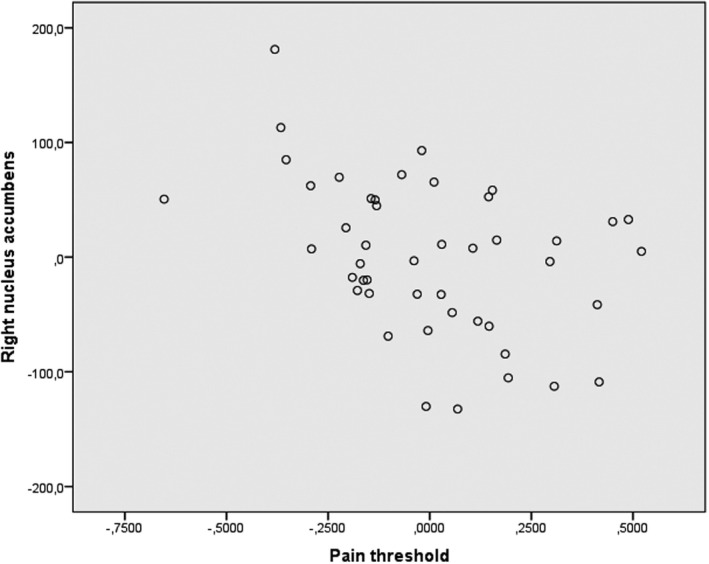
Association between the rectal pain threshold and the volume of right nucleus accumbens. Partial regression scatter plot illustrating the association between the rectal pain threshold transformed using natural logarithm and the volume of nucleus accumbens, controlled for age and TGMV. The *x*‐axis represents the residuals from predicting pain threshold from age and TGMV. The *y*‐axis represents the residuals from predicting right nucleus accumbens volume from age and TGMV

**FIGURE 4:**
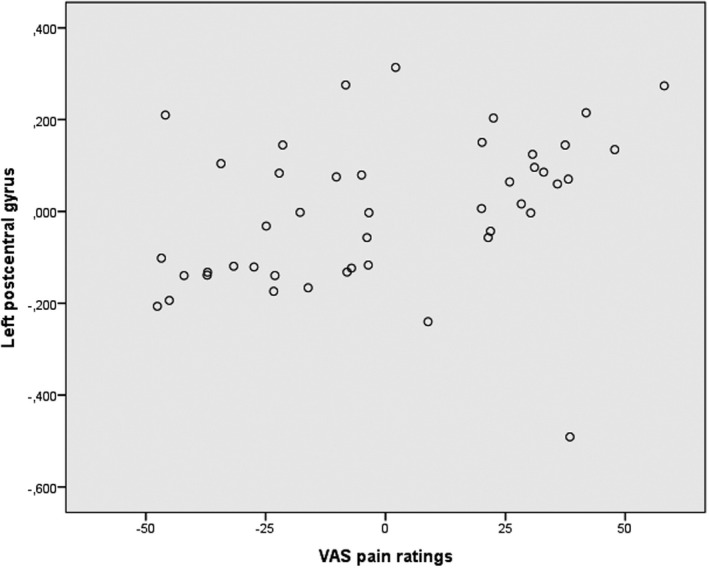
Association between pain ratings during rectal distension and cortical thickness of left postcentral gyrus. Partial regression scatter plot illustrating the association between the pain ratings at 24 mm Hg above BOP rectal distension and the cortical thickness of the left postcentral gyrus, controlled for age and TGMV. The *x*‐axis represents the residuals from predicting pain intensity ratings at 24 mm Hg from age and TGMV. The *y*‐axis represents the residuals from predicting left postcentral gyrus cortical thickness from age and TGMV

In IBS patients, increased volume of right nucleus accumbens was associated with lower rectal pain thresholds (*r* = −0.42, *p*_Bonferroni‐corrected_ = 0.025) (Figure 3), and higher pain ratings at 24 mm Hg were associated with higher cortical thickness of left posterior central gyrus (S1) (*r* = 0.45, *p*_Bonferroni‐corrected_ = 0.015) (Figure 4). At the uncorrected significance level, the pain ratings were also associated with the cortical thickness of right posterior central gyrus (S1) (*r* = 0.37, *p* = 0.014), and of the left superior part of the precentral sulcus (M1) (*r* = 0.31, *p* = 0.042), but these associations were not significant after correction for multiple comparisons.

### 3.5 Dataset 2: Correlations between sensorimotor regions and reported symptoms

In IBS patients, there were no correlations between brain morphometry of the sensorimotor network and reported severity of abdominal pain and bloating (GSRS) or psychological distress (HADS) that remained significant after Bonferroni correction. At the uncorrected significance level, there was a positive correlation between right nucleus accumbens volume and severity of abdominal pain (*r* = 0.35, *p* = 0.02) and bloating (*r* = 0.33, *p* = 0.027), and a negative association between psychological distress and left S2 cortical thickness (*r* = −0.31, *p* = 0.039).

## 4 DISCUSSION

Differences in regions of the sensorimotor network were identified in the biggest cohort published to date comparing regional gray matter properties between female IBS patients and HCs. Higher gray matter measurements were observed in primary somatosensory cortex, secondary somatosensory cortex, and subcortical regions, as well as lower gray matter measurements in posterior insula and superior frontal gyrus in IBS compared to HCs. Some of these differences were associated with measures of visceral perception in IBS. Specifically, correlations between pain intensity ratings during rectal distension and left primary somatosensory cortex, and between rectal pain thresholds and right nucleus accumbens were identified. Our results show that the central nervous system involvement in IBS is reflected in regional gray matter measurements and that the structural representation of visceral sensitivity in IBS involves a region with sensory‐discriminatory functions and a region important for pain chronification.

### 4.1 Gray matter morphometry measurements as indicators of neuroplasticity

Neuroplasticity refers to structural and functional changes in neural circuits in response to experience.^28^, ^29^ Experiences and learning can cause regional gray matter alterations identifiable with MRI.^29^, ^30^ There is mounting evidence for structural plasticity and reorganisation in human chronic pain in general,^31^, ^32^, ^33^, ^34^, ^35^, ^36^ as well as in IBS specifically.^10^, ^12^, ^37^, ^38^, ^39^, ^40^ The cellular correlates that underlie the macroscopically detected gray matter plastic changes are so far incompletely understood. They are thought to be composed of a combination of adult neurogenesis (for certain regions such as the hippocampus), synaptic changes (such as changes in dendritic length and branching or in the number of dendritic spines per neuron), and changes in the number and morphology of glial cells.^30^ It could also reflect a change in cell sizes, as well as changes in blood flow or interstitial fluid.^29^ In one study of fibromyalgia using multimodal neuroimaging, gray matter decrease was not evidently related to neurodegeneration, but possibly to reduced blood flow, whereas increased gray matter volume was associated with increased GABA_A_‐receptor concentration and water content, indicative of neural plasticity.^41^ The correlations with visceral sensitivity in this study were in regions with increased gray matter in IBS, which using the results of Pomares et al^41^ supports the interpretation of neural plasticity underlying these findings.

### 4.2 Group differences between IBS and HCs

The major group differences between health and IBS in this study were gray matter increases in primary somatosensory cortex, secondary somatosensory cortex and subcortical regions, as well as decreases in posterior insula and superior frontal gyrus in IBS patients (see Table 4). Postcentral gyrus has in several studies been described as being larger in IBS than health,^10^, ^12^, ^14^ and posterior insula smaller in IBS.^12^, ^14^, ^37^ Three of these studies were done using parts of the same cohort as the present study.^10^, ^12^, ^14^ However, this study is larger than previous studies, combining several cohorts and therefore contributes with increased robustness to the previous findings.

### 4.3 Associations between visceral pain sensitivity and brain structure

An abundance of research indicates that increased perception of visceral stimuli (visceral hypersensitivity) is a key component in IBS pathophysiology,^4^ but the structural central representation of this altered perception has not yet been established. In the current study, rectal pain thresholds and pain intensity ratings were associated with larger nucleus accumbens volume and primary somatosensory cortex thickness, respectively. This was in contrast to our hypothesis of correlations between visceral pain sensitivity measures and posterior insula. A previous report suggested that increased visceral sensitivity is associated with decreased gray matter volume in thalamus, cingulate, insula, amygdala, and basal ganglia in HCs.^15^ Several differences between the study by Elsenbruch et al and the current study prevent a direct comparison of the results. These differences include different definitions of pain threshold, different brain atlases, different statistical methods for correlational analyses, the Elsenbruch et al study used voxel‐based morphometry (VBM), and included both sexes. However, the differences found in the two studies suggest differences in brain morphometric associations with rectal sensitivity between IBS and HCs, which deserves further investigations in larger and combined samples of patients and HC using the same experimental setup.

#### 4.3.1 Primary somatosensory cortex

Primary somatosensory cortex (S1) has a role in pain processing, in particular regarding the localization of the stimuli,^42^ and it has been argued that neuronal activity in primary somatosensory cortex participates in producing awareness of the sensory/discriminative aspects of pain.^43^ There are also indications of primary somatosensory cortex being involved in modulation of the affective and attentional component of pain.^44^ Visceral stimuli, both innocuous and noxious, have been found to elicit activation in primary somatosensory cortex in HC, as well as in IBS subjects.^45^ In this study, IBS patients had increased cortical thickness of the primary somatosensory cortex compared to healthy controls, and we found an association between left primary somatosensory cortex thickness and rectal pain intensity ratings during rectal distension. This is in line with the notion that gray matter increase in primary somatosensory cortex is likely related to repeated exposure to pain.^46^

#### 4.3.2 Nucleus accumbens

The nucleus accumbens is important for motivational processes, reward, and avoidance, directing attention and behavior toward a goal or away from a threat.^47^ Nucleus accumbens is also involved in the transition from acute to chronic pain.^48^, ^49^ The increased volume of right nucleus accumbens in IBS shown in this study has not been reported before. In the second data set, the right nucleus accumbens was associated with rectal pain sensitivity, as well as reported severity of abdominal pain and bloating (albeit the latter two not significant after correction for multiple comparisons). Combining our results with the proposed role of nucleus accumbens in pain chronification, this might indicate that heightened visceral sensitivity might be related to pain chronification in IBS.

### 4.4 Associations between reported symptoms and brain structure in IBS

Contrary to previous studies^12^, ^14^ and our hypothesis, we did not find any significant associations between brain morphometry and reported severity of abdominal pain. Jiang et al^14^ found correlations with Bowel Symptom Questionnaire severity (BSQ) and bilateral pre‐ and postcentral gyrus in female IBS patients, and Labus et al^12^ found a positive correlation between volume of the left superior frontal gyrus and abdominal pain in IBS. There are several possible reasons for the discrepancies between these two studies and our study, including different assessment of abdominal pain (GSRS‐IBS vs BSQ) or different cultural settings (USA vs Sweden).

As hypothesized, there were no significant associations between sensorimotor regions and psychological distress. This is consistent with previous studies, where associations between psychological factors and gray matter morphometry in female IBS patients have mainly been seen in anterior and middle insula^14^ and prefrontal cortex.^50^ The lack of associations in the current study supports the notion that the results regarding visceral sensitivity is not related to psychological distress, but represents another pathophysiological mechanism evident at the central nervous system level, possibly neuroplasticity related to the experience of recurrent visceral pain.

### 4.5 Limitations

Hypersensitivity is present only in a portion of patients with IBS.^51^, ^52^ In this study, we did not differentiate IBS patients as normosensitive or hypersensitive, and it is likely that the associations between regional brain morphometry measures and visceral sensitivity measures would be different between these IBS subgroups. Future studies investigating correlations between visceral sensitivity and structural brain properties should consider taking the sensitivity status into account.

We did not include early life trauma or psychological distress in the group comparison, which in previous studies have been shown to influence the results.^12^, ^40^ However, our primary aim was to identify correlations with variables of visceral sensory processing and symptoms in brain regions of the sensorimotor network differing between IBS and HC, regardless of the potential influence of other parameters. Hence, we deemed it unnecessary to control for psychosocial factors in the group comparisons.

The correlational analyses in dataset 2 were performed in IBS patients only, not in healthy controls. It is therefore not clarified by this study if these correlations are specific to IBS or would also be seen in healthy controls. However, as previously discussed, the results from a study on healthy controls and visceral sensitivity^15^ showed results different from the results presented herein. The IBS subjects are patients seen at a secondary/tertiary care center for functional GI disorders, and the majority had severe IBS. Therefore, generalizing our results into IBS patients in other settings or with milder symptomatology is not possible without confirmatory studies.

Due to the cross‐sectional design, it is not possible to determine if the differences and associations seen in this study are causes or consequences of IBS and visceral hypersensitivity. Longitudinal studies suggest that the pain state drives the morphological changes, as opposed to morphologic alterations predisposing for the disease,^53^, ^54^, ^55^, ^56^, ^57^, ^58^ although no longitudinal gray matter studies have been published in IBS to date.

## 5 CONCLUSION

This study consolidates previous findings of gray matter alterations in IBS and contributes with new information about the central neurobiological substrate of visceral sensitivity in IBS, likely reflecting neuroplastic alterations. The structural representation of visceral sensitivity in IBS involves, at least, a region with sensory‐discriminatory functions and a region with motivational processes, important for pain chronification. Future studies of gray matter morphometry correlations with visceral sensitivity should include larger groups of both sexes, both IBS and HCs using the same visceral sensitivity testing protocol, and assess additional networks to obtain a more complete picture of these associations relevant for long‐standing GI symptoms.

## CONFLICT OF INTEREST

CG nothing to declare. HJR nothing to declare. LVO has received an unrestricted research grant from Nestlé (not related to the present work) and serves as a consultant for Danone. JSL nothing to declare. AP nothing to declare. ML nothing to declare. HT has served as Consultant/ Advisory Board member for Almirall and Allergan as a speaker for Tillotts, Takeda, Shire and Almirall. EAM is a member of Scientific Advisory Boards for Danone, Viome, Amare, Prolacta, Axial Biotherapeutics, Whole Biome, Ubiome, Bloom Bioscience and Mahana Therapeutics. MS received unrestricted research grants from Danone, Glycom and Ferring Pharmaceuticals and served as a Consultant/Advisory Board member for AstraZeneca, Danone, Nestlé, Almirall, Allergan, Albireo, Glycom, and Shire, and as a speaker for Tillotts, Menarini, Takeda, Shire, Allergan, Biocodex, Alimentary Health, AlfaSigma and Almirall.

## AUTHOR CONTRIBUTIONS

CG acquisition of data, analysis and interpretation of data, drafting of manuscript. HJR analysing and interpretation of data, drafting manuscript. LVO study concept and design, analysis and interpretation of data, critical revision. JSL acquisition of data, study concept and design, analysis and interpretation of data, critical revision. AP analysis and interpretation of data, critical revision. ML technical support, critical revision. HT acquisition of data, critical revision. EAM study concept and design, critical revision, study funding. MS study concept and design, critical revision, study funding.
